# Cataract surgery and artificial iris implantation in patient with oculocutaneous albinism: a case report

**DOI:** 10.5935/0004-2749.2022-0286

**Published:** 2024-03-27

**Authors:** Guilherme Vieira Peixoto, Gabriela Tomaz Martinho, Caio Cezar Toledo de Conti, Eduardo Villaça Filho, Renato Klingelfus Pinheiro

**Affiliations:** 1 Departamento de Oftalmologia, Irmandade da Santa Casa de Misericórdia de São Paulo, São Paulo, SP, Brazil; 2 Setor de Catarata, Departamento de Oftalmologia, Irmandade da Santa Casa de Misericórdia de São Paulo, São Paulo, SP, Brazil

**Keywords:** Cataract extraction, Albinism, oculocutaneous, Lens implantation, intraocular

## Abstract

We present a case report detailing the successful phacoemulsification surgery with artificial iris implantation for two individuals with oculocutaneous albinism. These women suffered from cataracts, resulting in reduced visual acuity and heightened photophobia due to iris pigmentary epithelium deficiency. The patients underwent phacoemulsification along with prosthetic artificial iris implantation into the posterior chamber. This intervention resulted in improved visual acuity, reduced photophobia and glare, and an overall enhanced quality of life. Our report highlights two cases of successful phacoemulsification and artificial iris implantation in patients with oculocutaneous albinism and cataracts, leading to improved visual acuity, reduced photophobia, and enhanced quality of life. Notably, there are no prior records in South American literature of cataract surgery combined with artificial iris implantation for oculocutaneous albinism patients up to the time of this publication.

## INTRODUCTION

Albinism is a hereditary condition characterized by reduced or absent melanin in hair, skin, and/or eyes due to inadequate production of the enzyme tyrosinase, which is essential for melanin synthesis. Individuals with albinism experience varying degrees of tyrosinase deficiency^(^1^)^.

Patients diagnosed with oculocutaneous albinism (OCA) exhibit reduced pigmentary epithelium in the iris and retina, accompanied by ocular issues like macular hypoplasia, nystagmus, and excessive retinostriate decussation^(^2^)^. This combination leads to diminished visual acuity, photophobia, and glare^(^3^)^.

Prosthetic iris implant surgery developed for patients with congenital, traumatic, or functional iris deficiencies can aid in visual recovery for individuals with iris deficiencies arising from OCA. This procedure can be conducted concurrently with or following cataract surgery^(^4^)^. While earlier studies have demonstrated favorable results in reducing photophobia and glare, the implantation of certain devices can pose surgical challenges^(^4^)^.

We present two cases of patients with OCA who underwent cataract surgery and artificial iris implantation, resulting in enhanced visual acuity and reduced photophobia and glare.

## CASE REPORTS

**Figure 1:**
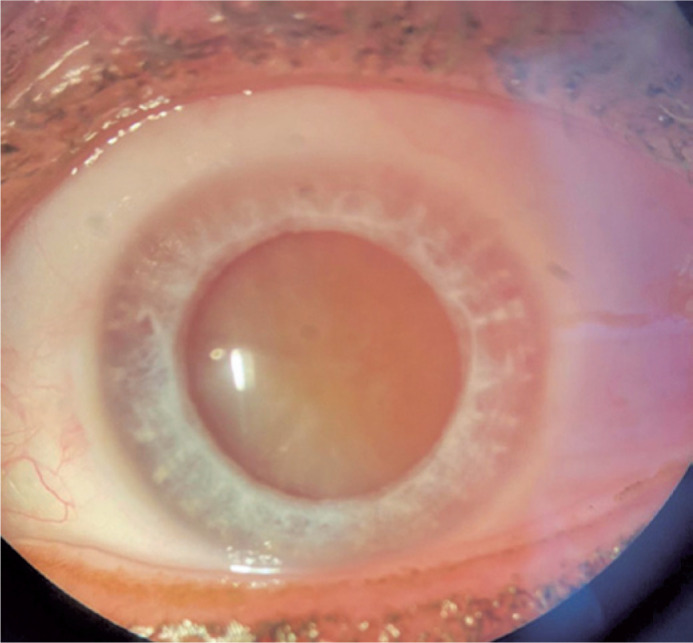
Intumescent total cataract on the left eye, prior to phacoemulsification.

The first patient is a 54-year-old woman diagnosed with OCA. She has experienced prolonged reduced visual acuity, which recently worsened in her left eye. She confirmed the absence of ocular trauma, existing medical conditions, ongoing medication use, or corticosteroid intake. A Snellen eye chart was employed to measure her best referred visual acuity, resulting in 20/200 for the right eye and hand-movement detection for the left eye. Detailed examination with a slit lamp revealed a marked deficiency in iris pigment and significant transillumination, both eyes grading 4+/4+ based on Kruijt et al.’s iris translucency classification^(^5^)^. The left eye exhibited an intumescent total cataract (Figure 1). Moreover, the fundoscopy for the right eye revealed inadequate retina pigment, grading 2+/3+ according to Kruijt et al.’s fundus pigmentation classification, along with macular hypoplasia^(^5^)^. However, fundoscopy of the left eye was impeded by crystalline opacity. Additionally, the patient had horizontal nystagmus.

Upon conducting ocular ultrasonography (USG) for the left eye, which revealed an anatomically healthy retina, the medical team performed phacoemulsification along with implant of intraocular lens (IOL). This procedure was combined with a prosthetic iris implantation onto the ciliary sulcus.

Initially, the surgical procedure commenced with phacoemulsification. Before the IOL implant was introduced, Trypan Blue was applied to the capsule. This was followed by the insertion of a standard polymethyl methacrylate (PMMA) endocapsular tension ring to avoid an asymmetrical contraction of the capsule, which could potentially lead to artificial iris dislocation. Subsequently, a spherical one-piece monofocal intraocular lens was implanted, followed by the placement of the iris. To determine its size, an average measurement was calculated using the white-to-white distance minus 0.5 mm according to Mayer et al.’s method^(^6^)^. The device was implanted within the posterior chamber (ciliary sulcus) after a trepanation using a corneal receptor trepan without an iridectomy. The artificial iris used is from the HumanOptics (Fiber-Free model), with 12.8 mm diameter (before trepanation) and fixed pupil (3.35 mm), and it sported a standard medium brown color along with an opaque black posterior surface to absorb light entry. This design choice aimed to minimize photic phenomena, enhance contrast sensitivity, and eliminate transillumination defects. Furthermore, the artificial iris was modeled based on the patient’s original iris, adhering to its natural structure and personal coloring without altering the inherent pigmentation.

**Figure 2:**
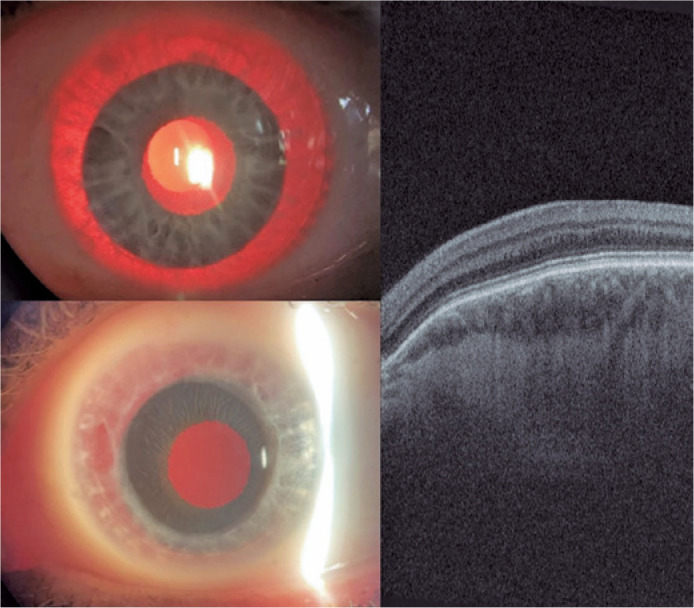
Left-eye slit lamp photograph 30 days after surgery, showing prosthetic iris on ciliary sulcus through retro illumination and after pharmacologic mydriasis. Retinal OCT showing absence of foveal pit and persistence of retinal inner layers through expected area of fovea (macular hypoplasia).

Initial assessment of visual acuity was not assessed during the early postoperative phase. However, after a span of 30 days following the surgery, a 20/150 visual acuity was achieved based on the Snellen eye chart. After the surgery, retinal optical coherence tomography (OCT) was performed, confirming macular hypoplasia in the operated eye (Figure 2).

The second patient, also a 54-year-old woman diagnosed with OCA, similarly reported prolonged diminished visual acuity which recently deteriorated in her left eye. Utilizing the Snellen eye chart, the best referred visual acuity was 20/80 for the right eye and recognition of hand movement for the left eye. Notably, she had undergone phacoemulsification for her right eye back in 2017.

**Figure 3:**
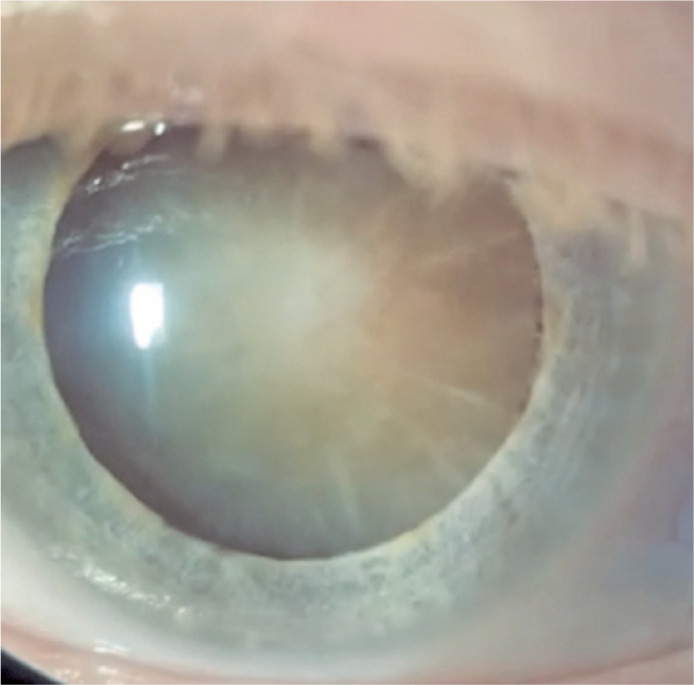
Intumescent total cataract on the left eye, before phacoemulsification.

Upon undergoing slit lamp examination, she demonstrated iris pigment deficiency and transillumination, with both eyes graded as 2+/4+ on Kruijt et al.’s iris translucency classification^(^5^)^. In the left eye, an intumescent total cataract was observed (Figure 3). During fundoscopy of the right eye, retina pigment deficiency was evident, grading 2+/3+ according to Kruijt et al.’s fundus pigmentation classification, as well as macular hypoplasia^(^5^)^. Owing to crystalline opacity, fundoscopy of the left eye could not be performed. She also presented horizontal nystagmus.

Once an anatomically healthy retina was observed through ocular USG, the surgical procedure moved forward to include phacoemulsification alongside IOL implantation. This was carried out concurrently with prosthetic iris implantation in the ciliary sulcus, following the same surgical protocol as applied for the first patient.

**Figure 4:**
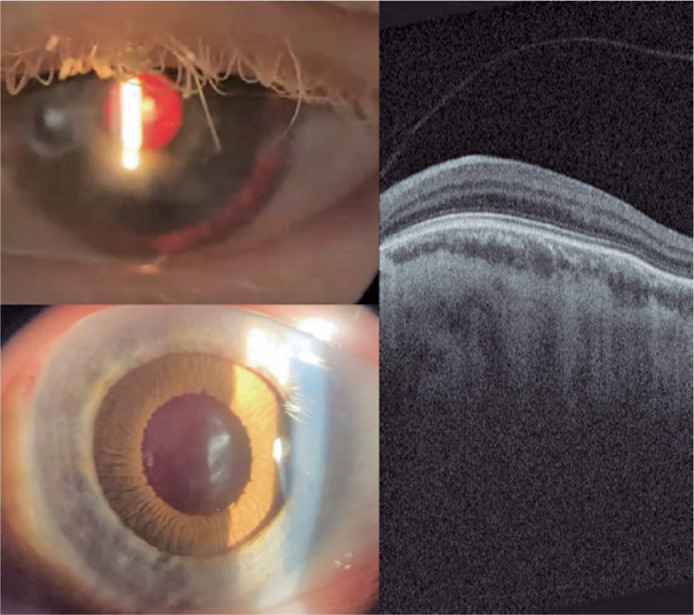
Left-eye slit lamp photograph 30 days after surgery, showing prosthetic iris on ciliary sulcus through retro illumination and after pharmacologic mydriasis. Retinal OCT showing absence of foveal pit and persistence of retinal inner layers through expected area of fovea (macular hypoplasia).

A month following the procedure, the operated eye exhibited an improved corrected visual acuity of 20/80. Subsequent to the surgery, retinal OCT was performed, confirming macular hypoplasia in the operated eye (Figure 4).

To assess photophobia, the Visual Light Sensitivity Questionnaire-8 (VLSQ-8) was utilized^(^7^)^. This questionnaire, designed to subjectively gauge photophobia, consists of eight questions pertaining to glare and photophobia symptoms. A comparison was drawn between pre-surgery and post-surgery responses. The first and second patients scored 25 and 30, respectively, regarding symptoms before the surgery, and these scores dropped to 15 and 8, respectively, after the intervention.

## DISCUSSION

Albinism comprises a collection of inherited conditions marked by diminished or absent melanin production^(^2^)^. The precise role of melanin in visual system development remains somewhat unclear, though its significance if evident. When pigment production is sufficiently reduced, regardless of the etiology, a stereotypic set of defects in neuronal migration within the visual pathway emerges^(^8^)^. Generally, the severity of melanin deficiency in the eye correlates with the severity of visual manifestations. Ocular alterations and symptoms encompass strabismus; positive angle kappa; photophobia; refractive errors; pendular nystagmus; reduced iris pigment leading to transillumination defects; foveal hypoplasia resulting in significantly reduced visual acuity, typically ranging from 20/60 to 20/400; and abnormal decussation of the optic nerve fibers^(^9^)^.

It is established that performing cataract surgery involving IOL implantation alone in patients with OCA, without addressing the iris pigment deficiency, can lead to relevant aberrations. This is due to the effect of the light at the IOL margin, potentially exacerbating functional visual disability^(^4^)^. Given that both patients had presented cataracts with reduced visual acuity, this would not have worsened their condition. However, if iris implantation had not been undertaken, these aberrations would appear, accompanied by photophobia and glare.

In individuals without OCA, visual photosensitivity serves as a physiological defense mechanism against potentially harmful intense light (e.g., sunlight). While those without OCA general possess high tolerance to bright light, many conditions, including ophthalmic disorders like ocular albinism, can elevate visual photosensitivity, detrimentally affecting one’s quality of life^(^10^)^.

Significant enhancements in visual acuity were notable, transitioning from perceiving hand movements to 20/150 in one case and to 20/80 in the other, as determined through the Snellen eye chart. A marked positive influence was observed in relation to photophobia and glare. The scores obtained from the VLSQ-8 indicated symptom severity scores of 25 and 30 before the surgery, which reduced to 15 and 8 subsequently. Both patients exhibited improvement regarding photophobia frequency and severity, along with diminished impact on activities like reading and utilizing television and computers. In the case of the second patient, an improvement in headaches associated with photophobia was described. Therefore, both cases experienced important improvement concerning photophobia and glare, as reported by the patients during the follow-up.

In conclusion, we successful influenced the patient’s quality of life by improving visual acuity and reducing light-related symptoms and aberrations. This achievement was made possible through a surgically intricate procedure that was carried out without complications during both the operation and subsequent follow-up. Until the time of publication, no other literature has documented cases of cataract surgery coupled with artificial iris implantation for individuals with oculocutaneous albinism in South America.

### Link for Surgery Video

https://youtu.be/n6g03URFuYc

https://youtu.be/1y-3g_I3yXg
